# Using social psychology to create inclusive education

**DOI:** 10.1111/bjso.12867

**Published:** 2025-02-13

**Authors:** Matthew J. Easterbrook, Lewis Doyle, Daniel Talbot

**Affiliations:** ^1^ School of Psychology University of Sussex Brighton UK; ^2^ Centre de Recherches sur la Cognition et l’Apprentissage (CeRCa) Université de Poitiers Poitiers France; ^3^ Thomas Tallis School London UK

**Keywords:** education, identity, inequality, interventions

## Abstract

Social psychological processes related to identities and stereotypes—such as threat, belonging uncertainty, identity incompatibility and bias—can be ignited by features and practices in educational contexts, often further disadvantaging members of minoritised or underrepresented groups. Such psychological processes are consequential and predict hard academic outcomes such as attainment and progression. Although this knowledge can be harrowing, it also gives us the power to intervene. We propose three ways in which social psychology can be used to help create more inclusive education systems: by using interventions wisely, working with teachers to collaboratively create inclusive classrooms, and by fighting bias. We offer concrete examples of how social psychology is helping to reduce educational inequalities in these ways, as well as some suggestions for the future.

Inequalities in educational outcomes are ubiquitous across the Globe. In England, where we^1^ are based, boys, students from certain ethnic groups, and especially students from low‐income families have – on average – poorer educational outcomes than their peers. These inequalities are large and seemingly intractable. Despite billions of pounds of Government funding being invested into reducing the socioeconomic attainment gap in England in recent decades, it is estimated that it would currently take over three years' worth of additional teaching to help 16‐year‐olds from low‐income families (i.e. those eligible for free school meals) to reach the same level of attainment as their peers (Easterbrook & Hadden, 2021), a gap that is widening in the aftermath of the pandemic (Goudeau et al., 2021). This is not a problem that is unique to England; countries across the world also face persistent educational inequalities, although the size and groups involved vary (Enchikova et al., 2024). Traditional educational interventions – such as smaller classes, or new facilities or teaching techniques – are expensive and often have limited impact (Kraft, 2019). As purse strings tighten around already underfunded education systems, the time is apt for a new approach to reducing inequalities, one that harnesses the power of social psychology.

We draw on classic social psychological theories – including social comparison theory (Festinger, 1954), social identity theory (Tajfel & Turner, 1979), possible selves theory (Oyserman & James, 2011) and implicit social cognition (Greenwald & Banaji, 1995) – to help us understand the psychology that contributes to inequalities in educational outcomes. Below, we draw on ours and others' research, as well as our own programme of engagement work with schools, to offer some insights into how social psychology can be practicably used to reduce inequalities in educational outcomes.

## SOCIAL PSYCHOLOGY IN EDUCATION

As people try to decide what life path to pursue, they often ask themselves fundamental questions such as ‘Who am I?’ and ‘What might I be good at?’. In the absence of direct answers, these questions necessarily become social in nature: ‘Who are we?’, ‘What have people like me done in their lives?’, ‘Where are people like me represented, wanted, and valued?’. The answers to these questions are derived from looking to those we know and identify with most strongly; our family, community, and neighbours, that is, ‘people like us’ (Dasgupta, 2011; Elmore & Oyserman, 2012; Oyserman et al., 2006). For those who live in under‐resourced neighbourhoods where few people have had positive experiences in education or hold jobs requiring higher educational qualifications, there may be few reasons to believe education is a worthwhile endeavour for people like you.

This is not an inconsequential belief. We have found that students from low‐income families in England are less likely to agree with statements such as ‘people like me tend to do well at school’. Those who disagreed with such statements – those with high levels of *identity incompatibility* – had worse exam grades at age 16 (Easterbrook et al., 2022), applied to lower‐ranked universities than their grades afforded (Nieuwenhuis et al., 2019), and performed worse at university (Rimicci et al., in press). Research in the US (Herrmann et al., 2022) and in several EU countries (Krpanec et al., 2023; Matschke et al., 2022; Veldman et al., 2019) have documented similar identity processes that contribute to the educational experiences of those from low‐income families. Identity incompatibility, then, is not a woolly or soft concept that educational policy makers can overlook; it predicts so‐called ‘hard’ academic outcomes and helps explain socioeconomic inequalities in educational outcomes.

Importantly, we also found that low‐income students in England had much lower levels of identity incompatibility—equal levels to their peers, in fact—and so better grades if they were in schools in which people like them—other low‐income students—had done well in the past (Easterbrook et al., 2022). This shows how the local social and cultural contexts inform how we construct our identities, that more unequal schools and societies can ignite psychological processes that drive inequality (Claes et al., 2024) and, crucially, that schools and societies can create inclusive and safe environments that enable low‐income students to thrive.

Yet, schools are often fighting against wider society. In England, for example, where over half the population identify as working class (Spurdens & Crabb, 2024), just 4% of doctors and only 10% of Oxford and Cambridge undergraduates are from working‐class backgrounds (Social Mobility Commission, 2016; The Sunday Times, 2018). This underrepresentation of working‐class individuals in elite positions and institutions is common internationally (e.g. Bovens & Wille, 2017). Although few people are probably explicitly aware of statistics like these, they form part of the social fabric of society and act as tacit messages about whom we should expect to do what: that is, our identities and possible selves, but also our stereotypes, prejudices and biases (Eagly & Koenig, 2021; Oyserman & James, 2011).

Teachers are as susceptible to stereotypes and biases as the rest of us (Starck et al., 2020). In England and Western Europe, such biases tend to reinforce existing socioeconomic inequalities. For example, trainee teachers in Germany associate competence and good educational habits with high socioeconomic status (SES; Glock et al., 2013), and, in England, trainees display a preference against working in schools that serve under‐resourced communities (Doyle & Easterbrook, 2023). In French preschools, teachers are less likely to call on students from lower compared to higher‐class backgrounds in classroom discussions, meaning lower‐class students have fewer opportunities to contribute and practice oracy (Goudeau et al., 2023). In Switzerland, teachers tend to believe a lower‐ability educational track is more appropriate for students from low‐ versus high‐income families, even when their academic ability is identical (Batruch et al., 2018). In England, teachers have also been found to give a piece of written work a lower grade, perceive the student who wrote it to be working at a lower level, and suggest the student should be placed in a lower‐ability group if they believed the work was written by a student from a low‐ compared to higher‐income family (Doyle et al., 2022; see Batruch et al., 2017 for similar work from Switzerland).

Perhaps unsurprisingly given these results, students from low‐income families are more likely to feel threatened and that they do not belong in educational settings (Croizet & Claire, 1998; Harackiewicz et al., 2014; Spencer & Castano, 2007). Experiencing threat disrupts learning (Grand, 2016), persistence and performance (Walton et al., 2012; Walton & Cohen, 2007), and makes students less likely to fully engage in education or take advantage of the support and resources on offer. Perhaps most concerning is that threat can be ignited by commonplace classroom practices such as hand raising (Goudeau & Croizet, 2017), receiving communications that use unfamiliar wordings (Stephens et al., 2012) or seeing stereotypical posters (Cheryan et al., 2009).

The above overview can make for uncomfortable reading, especially for teachers, who are genuinely motivated to support and educate all their students. But the evidence should give us hope, too. Understanding the psychology that can contribute to inequalities in educational outcomes gives us the power to intervene and create more inclusive classrooms. So, armed with such knowledge, what can we do to address educational inequality?

## RECOMMENDATIONS

### Use interventions wisely

First, we can use a range of brief so‐called *wise* interventions to support students (Walton, 2014). Wise interventions target specific, self‐relevant processes or perceptions that are experienced by specific groups of students – usually those from stereotyped or stigmatized groups – in ways that can reduce their engagement and performance. By changing these processes or perceptions, wise interventions aim to remove psychological barriers that certain groups of students face so that they have the freedom to achieve their potential (Walton, 2014).

Wise interventions can be remarkably effective at reducing inequalities in educational outcomes. For example, we (Hadden et al., 2020) implemented a self‐affirmation intervention—which is thought to reduce the impact of threat on educational performance (Easterbrook et al., 2021)—in a school in England, and found it closed the socioeconomic attainment gap in end‐of‐year mathematics tests by 62%, with some effects sustained for at least 4 years (Hadden et al., 2024). Other *wise* interventions—including social belonging (Walton & Brady, 2020), difference‐education (Stephens et al., 2015), empathic discipline (Doyle et al., 2024; Okonofua et al., 2016) and revealing hidden similarities between teachers and students (Gehlbach et al., 2016; Hadden, Harris, & Easterbrook, 2025)—have found equally impressive effects (see Easterbrook & Hadden, 2021, for a review).

Yet, when such interventions are implemented in contexts in which the psychological processes they target are not contributing to inequalities, they can be ineffective and an inefficient use of resources (Easterbrook & Hadden, 2021). For example, a much larger self‐affirmation trial we implemented across 29 schools (See et al., 2022) found a much weaker average effect of self‐affirmation for free‐school meal‐eligible students, presumably because threat was not contributing to educational inequalities in all of the schools.

To intervene effectively, then, we need to understand the specific challenges faced by students in the context in which we're working. This is the premise behind the identities‐in‐context model of educational inequalities (Easterbrook et al., 2019) and accompanying protocol (Easterbrook & Hadden, 2021) designed to help educational practitioners choose an intervention likely to be effective at reducing inequalities in their school. The protocol has three simple steps. First, identify what the inequalities in the school are that you want to reduce. Second, conduct as much background work as possible to understand what, if any, social, cultural and/or psychological factors are contributing to these inequalities. Finally, consider the resources available and choose (and evaluate) an intervention that targets those precise processes.

We have now published our first implementation and evaluation of this approach (Hadden et al., 2024). Our diagnostic work in the participating schools identified poorer teacher–student relationships and a sense of threat were contributing to lower attendance and poorer behaviour for Black (compared to Asian) students and for low‐ (compared to higher‐) income students. To target these psychological processes, we implemented a mix of values affirmation (to target threat) and revealing hidden similarities (to develop teacher–student relationships; Gehlbach et al., 2016). Although the intervention was heavily disrupted by the COVID‐19 pandemic, it reduced the socioeconomic attendance gap by over 60% and had positive—though non‐significant—trends on the other gaps. What is more, it was more effective for students who had poorer relationships with their teachers and were more threatened at baseline; the precise psychological processes we targeted.

Although targeted psychological interventions can be impressively effective, it is vitally important to be aware and to communicate that they are a sticking plaster, not solution (Blanton & Ikizer, 2019; Chater & Loewenstein, 2023; Ikizer & Blanton, 2016). They may help a small number of students, but the conditions that gave rise to the inequalities will remain.

We need Government initiatives that address structural, economic and historical injustices if we are serious about reducing educational inequalities. Yet, reducing such injustices is a long‐term goal that, even if proactively pursued by governments, is unlikely to bring about any meaningful change for many currently in the education system. We therefore also need shorter‐term actions that support underserved and minoritised groups to achieve their potential within an unjust and discriminatory system (Tucker & Herman, 2002). That is what these interventions can offer.

As more evaluations of interventions are conducted, we strongly encourage researchers to document and, where possible, analyse contextual factors that may moderate the effectiveness of interventions (Goudeau et al., 2024). This will enable researchers to continue to develop theories that outline when and where interventions are likely to be most effective (Easterbrook & Hadden, 2021), offering insights into how to create more inclusive contexts and thus reduce the need for short‐term interventions.

### Collaboratively create inclusive classrooms

Simultaneously, we can go upstream and work collaboratively with teachers to make classrooms as inclusive and bias‐free as possible. Drawing on co‐production strategies (Verschuere et al., 2012), we are holding workshops with teachers in English schools in which we explain the psychology of threat, inclusion and wise interventions in a school‐specific way. This enables and empowers teachers to take action to create more inclusive classrooms (School Inclusion Group, n.d.).

For example, there are several common school practices that can be threatening for stigmatized students, including hand raising (Goudeau & Croizet, 2017) and ability grouping (Hamilton & Hara, 2011). Armed with the knowledge of how and why these practices can ignite threat and disadvantage some students, teachers at the schools we are working with have eliminated these practices as much as possible. Other schools have developed novel ways of implementing wise interventions in their classrooms. For example, several schools have translated social belonging interventions (Walton & Brady, 2020) into new, accessible formats (e.g. videos) designed to support students through school transitions. These co‐developed policy and practical developments also feed back into research, as we are now designing ways to evaluate their effects in situ.

The schools we are working with serve a range of different types of communities. Some are ethnically homogeneous but are grappling with inequalities based on gender or SES. Others serve extremely diverse communities and have large subgroups of students from refugee backgrounds. No single psychological process is contributing to educational inequalities in these schools, and no single intervention will be universally effective. In fact, the only point relevant to all the schools we are working with is that each school is different: they have different groups of students, different inequalities, and there are different psychological processes that contribute to these inequalities. Yet, increasing understanding about how psychology can contribute to inequalities, and providing tools designed to help create safe and inclusive classrooms, can empower teachers to work towards reducing inequalities by reducing threat and fostering feelings of belonging, respect and inclusion among their students. Again, these approaches fuel further research into their effectiveness at engaging teachers and supporting students.

These are examples of how knowledge exchange and co‐production can help translate social psychological theory and research into concrete pedagogical approaches for schools, and how this can fuel further research.

### Fight bias

Although teachers recognise that bias is a problem in education, they often find it challenging to acknowledge their own biases (Doyle et al., 2024). Yet, as becoming aware of biases is an important first step towards reducing them (Devine et al., 2012), it is vital that the potential for bias in education is understood and recognized by teachers.

Training materials that emphasize structural over individualistic causes of inequality have the potential to increase teachers' awareness of biases and so their power to reduce them (Doyle & Easterbrook, 2023). Initial teacher education providers do sometimes incorporate such information into their teacher training models, but the extent and quality appear to vary widely across courses (ibid.). Creating space to learn about and reflect on socioeconomic biases, as well as those relating to other legally protected characteristics like race and gender (Rickett et al., 2022), should be a mandatory and important part of teacher training.

Teachers also seem better able to monitor and attenuate their biases when they have the time and cognitive capacity to do so (Doyle et al., 2024). Although this is rarely possible in chronically underfunded education systems, head teachers and policy makers should strive to develop working conditions that are conducive to de‐biased classrooms. Simple tools such as marking rubrics or anonymising assessments can also effectively help reduce bias (Andrade, 2005; Quinn, 2020) and should be utilized in as many areas as possible, including for social interactions and classroom discussions.

Providing tools such as rubrics that help identify and reduce bias, offering compelling narratives that emphasize structural over individual attributions for academic performance (Hadden, Darnon, et al., 2025), and offering opportunities to have meaningful contact with people from different groups (Doyle et al., 2024) can all help teachers begin to identify and reduce their biases. Schools can initiate discussions with teachers that encourage perspective taking and offer counter‐stereotypical narratives about their students, and should support teachers to create inclusive classrooms by showcasing counter‐stereotypical images and celebrating the achievements of individual students (Devine et al., 2012). Reducing bias is a process that takes time and institutional support, but one that is crucial to reducing educational inequalities.

## CONCLUSION

In sum, we have argued that a range of social psychological processes contribute to the concerning inequalities plaguing education systems across the globe. Not only can this understanding inform public thought and debate on the issues of social justice in education, but with the engagement and support of educators and policymakers, social psychology can also be used to help directly combat these inequalities. In particular, we have advocated intervening wisely, collaboratively creating inclusive classrooms, and fighting bias as three areas where social psychology can directly help reduce inequalities in educational outcomes.

## AUTHOR CONTRIBUTIONS

**Matthew J. Easterbrook:** Conceptualization; funding acquisition; investigation; writing – original draft; project administration; resources. **Lewis Doyle:** Writing – review and editing. **Daniel Talbot:** Writing – review and editing.

## CONFLICTS OF INTEREST

There are no conflicts of interest.

## Footnotes

^1^
In this article, we use ‘we’ to refer to the members of our research group, both past and present.
